# Shared Genetic Links Between Nonalcoholic Fatty Liver Disease and Coronary Artery Disease

**DOI:** 10.5334/gh.1374

**Published:** 2024-11-26

**Authors:** Hua Di, Shouhao Wang, Chengan Xu, Qiaoqiao Yin, Keyang Xu, Wei Zheng

**Affiliations:** 1 Geriatric Medicine Center, Department of Acupuncture & Massage, Zhejiang Provincial People’s Hospital (Affiliated People’s Hospital), Hangzhou Medical College, Hangzhou 310014, Zhejiang, China; 2 Hepatology Diagnosis and Treatment Center, The First Affiliated Hospital of Wenzhou Medical University and Zhejiang Provincial Key Laboratory for Accurate Diagnosis and Treatment of Chronic Liver Diseases, Wenzhou 325035, Zhejiang Province, China; 3 Center for General Practice Medicine, Department of Infectious Diseases, Zhejiang Provincial People’s Hospital (Affiliated People’s Hospital), Hangzhou Medical College, Hangzhou, Zhejiang 310014, China; 4 Centre for Cancer & Inflammation Research, School of Chinese Medicine, Hong Kong Baptist University, Hong Kong, China

**Keywords:** Nonalcoholic fatty liver disease, Coronary artery disease, Genetic association study, Mendelian randomization analysis, Genetic pleiotropy

## Abstract

**Background:**

Epidemiological and clinical studies have shown that there is a co-morbidity between nonalcoholic fatty liver disease (NAFLD) and coronary artery disease (CAD).

**Methods:**

In this study, we utilized linkage disequilibrium score regression (LDSC) to evaluate the genetic correlation between non-alcoholic fatty liver disease (NAFLD) and coronary artery disease (CAD). We identified pleiotropic loci and genes using SNP-Level PLACO analysis. Following this, MAGMA gene set enrichment analysis was conducted to assess the biological significance of these pleiotropic genes. Finally, a two-sample two-way Mendelian randomization (MR) analysis was performed to evaluate causal relationships between NAFLD and CAD.

**Results:**

We found a significant genetic correlation between NAFLD and CAD. Secondly, PLACO multi-effect analysis identified 6 sites (mainly involved in the establishment of chylomicrons, mitochondrial membrane protein localization and herpes simplex virus 1 infection signaling pathway). Then, three pleiotropic genes (APOC1, TOMM40 and PBX4) were identified by MAGMA gene analysis. Finally, a two-sample two-way MR analysis suggested that there was no causal relationship between NAFLD and CAD.

**Conclusions:**

Our results show that there are significant gene overlaps and pleiotropic genes between NAFLD and CAD and point out their common molecular mechanisms. These findings provide evidence for the common etiology between them and also help to better understand the pleiotropic nature between NAFLD and CAD, which may be of guiding significance for future treatment strategies.

## Introduction

Nonalcoholic fatty liver disease (NAFLD) is one of the liver manifestations of metabolic syndrome (MS) and an important global public health problem (1,2). NAFLD can develop into liver fibrosis, cirrhosis, and even hepatocellular carcinoma (HCC).

However, although these liver-related outcomes are worrying, the most common cause of death in patients with NAFLD is cardiovascular disease (CVD) (3), especially coronary artery disease (CAD) (4). At present, many epidemiological and clinical studies have focused on the relationship between NAFLD and CAD (5). The occurrence of NAFLD and CAD is the result of the interaction of multiple genetic variations and environmental factors, although its pathogenesis has not been fully elucidated (6,7). The common pathogenesis of NAFLD and CAD includes insulin resistance (IR), atherosclerotic dyslipidemia, subclinical inflammation, oxidative stress, etc. (6,8).

Studies have found that genetic polymorphisms can affect individual susceptibility to NAFLD and CAD (9,10). The genomic characteristics of these two diseases have been widely studied. Among them, peroxisome proliferator-activated receptors gene (PPAR) (11), leptin receptor (LEPR) (12,13), apolipoprotein C3 (APOC3) (14,15) and so on have been reported to be associated with NAFLD and CAD (6). Therefore, further research on the correlation between NAFLD and CAD has attracted much attention.

In this study, we investigated the genetic association between NAFLD and CAD. Previous studies have shown that there may be a link between these two diseases, and their common genetic background may involve pleiotropic effects (16,17,18). Our aim is to explore this genetic overlap by using the linkage disequilibrium score regression (LDSC) method for GWAS data of NAFLD and CAD. This approach allows us to identify pleiotropic loci and genes, which may shed light on potential sharing mechanisms. In addition, we performed functional enrichment analysis to characterize the relevant biological pathways and used Mendelian randomization (MR) analysis to evaluate causal relationships. These methods together support our effective treatment of NAFLD and CAD on the basis of gene-based treatment strategies.

## Methods

### Data sources

The NAFLD data was from FinnGen R9 GWAS (https://r9.finngen.fi/). The data included 2,275 cases and 375,002 controls. FinnGen collected and analyzed genomic and health data from 500,000 Finnish biobank participants. On the one hand, it provides novel medical and treatment-related insights while also building world-class resources that can be used for future research.

The CAD data was from a GWAS meta-analysis (19). This study performed a genome-wide association study of 34,541 CAD cases and 261,984 controls from the UK Biobank database and replicated in 88,192 cases and 162,544 controls of CAD. A total of 75 reproducible and genome-wide significances were identified in the meta-analysis.

### Genetic correlation analysis

The linkage disequilibrium score regression (LDSC) method is used to evaluate the shared multi-gene structure between traits (20). The LD score in LDSC can be calculated from the thousand-person genome as a reference group and the European blood samples in the Hapmap3 project (21). For SNPs, we implemented strict quality control: (i) excluding non-biallelic SNPs and those with chain fuzzy alleles; (ii) exclusion of SNPs without rs tag; (iii) repetitive SNPs or SNPs not included in the 1000 Genome Project or whose alleles did not match were deleted; (iv) due to its complex LD structure, SNPs located in the major histocompatibility complex (chr6: 28.5–33.5 Mb) region were excluded in LDSC analysis and (v) SNP with minor allele frequency (MAF) > 0.01 was retained.

### Pleiotropic analysis

*SNP-Level* PLACO is a new method to study pleiotropic loci between complex traits using only aggregated genotype-phenotype association statistics (22). In our analysis, we first calculated the square of the Z score for each genetic variant to assess their contribution to the traits of interest. To ensure data integrity, we removed SNPs with extremely high Z² values (Z² > 80), which could indicate outliers or erroneous associations. In addition, considering the potential correlation between NAFLD and CAD, we estimated the correlation matrix of Z. We applied a threshold of 5E-8 for statistical significance. Subsequently, we tested the null hypothesis using the implementation under test (IUT) method, which assesses the maximum p-value derived from both the null hypothesis (H_0_) and the alternative hypothesis (H_1_). This rigorous approach allowed us to robustly identify loci that may contribute to the pleiotropic relationship between NAFLD and CAD.

Based on the PLACO results, we further mapped the identified loci to nearby genes to explore the common biological mechanism of these pleiotropic loci. We performed Generalized Gene-Set Analysis of GWAS Data (MAGMA) analysis on genes located at or overlapped with pleiotropic loci based on PLACO output and single-trait GWAS to identify pleiotropic candidate pathways and tissue enrichment of pleiotropic genes (23). To identify candidate pleiotropic genes, the significance of MAGMA analysis was *P* < 0.05 / N_genes_ = 3E-06. The significance of MAGMA pathway and tissue analysis was corrected by Bonferronni correction. The functional maps and annotations (FUMA) of genome-wide association studies are used to determine the biological functions of pleiotropic loci (24). At the same time, based on the molecular signatures database (MSigDB), a series of pathway enrichment analyses were used to determine the function of mapped genes (25). The eQTL analysis included SNP-gene association data, including whole blood tissue.

### Mendelian randomization study

We used the clumping program in PLINK software to screen out all significant gene loci independently associated with disease as instrumental variables (*P* < 5 × 10^–8^). The r^2^ threshold of the instrumental variable was set to 0.001, and the window was set to 10000kb (26). In order to ensure the strength of the instrumental variables, we calculate the r^2^ and F statistics of each instrumental variable (27). The F statistic is calculated as follows, \[
F\,{ = }\,(\frac{{n\,-\,1\,-\,k}}{k})(\frac{{{r^2}}}{{1\,-\,{r^2}}})
\] , where *r*^2^ represents the proportion of variance explained by the instrumental variable, *n* represents the sample size, and *k* represents the number of SNPs. The main method used in Mendel’s randomization is inverse variance weighting (IVW), which requires the instrumental variable (IV) to meet three assumptions: (1) IV should be related to exposure; (2) IV should not be associated with confounding factors associated with exposure and outcome and (3) the effect of IV on the results was achieved entirely through exposure. We conducted several sensitivity analyses. First, the Q test using IVW and MR-Egger can detect potential violations of the hypothesis through the heterogeneity of the association between each IV (28). Secondly, we applied MR-Egger to estimate horizontal pleiotropy based on its intercept to ensure that genetic variation is independently associated with exposure and outcome (29). We increase the stability of the results by using additional analysis (weighted medians and weighted patterns) of MR methods with different modeling assumptions and advantages.

Statistical analysis was performed using R 3.5.3 software. MR analysis was performed using the Mendelian Randomization software package (30).

## Results

### A significant genetic correlation between NAFLD and CAD

Genetic correlation analysis showed that there was a significant genetic correlation between NAFLD and CAD, whether in the analysis of LDSC containing the intercept term (r_g_ = 0.439, *P* = 2E-04) or in the analysis of limiting the intercept term to 0 (r_g_ = 0.322, *P* = 2.77E-09).

### Three pleiotropic genes between NAFLD and CAD

**Figure 1:**
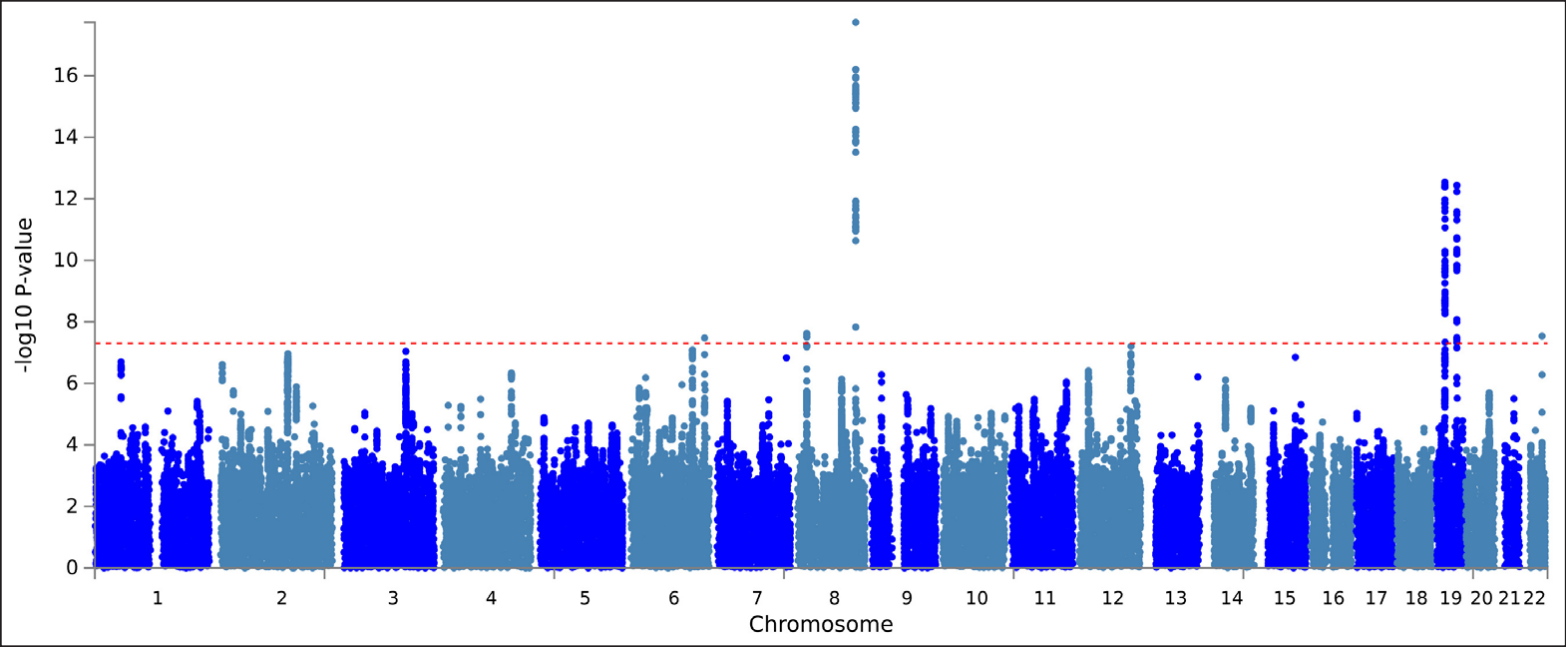
Manhattan diagram of pleiotropic loci between CAD and NAFLD.

PLACO pleiotropic analysis was further performed on NAFLD and CAD. A total of 6 pleiotropic genomes were identified, which were 6q25.3, 8p21.3, 8q24.13, 19p13.11, 19q13.32 and 22q13.31, respectively. The Manhattan diagram is shown in Figure 1, and the pleiotropic loci identified are shown in Table 1. No genomic inflammation was found in the QQ plot (Figure S1), and the basic information of each genomic risk locus is shown in Figure S2. The effect of pleiotropic SNP on gene function is shown in Figure S3. The regional plot of each risk locus is shown in Figures S4–S9.

**Table 1:** Information of 6 pleiotropic loci identified.


GENOMIC LOCUS	UNIQID	CHR	START	END	LEAD SNPS	P–VALUE	MAPPED GENES	PP H4

6q25.3	6:160499565:C:T	6	160067581	160929904	rs2297362	3.32E–08	IGF2R	0.282

8p21.3	8:19942908:A:C	8	19774890	19956346	rs11986461	2.37E–08	AC100802.3	0.590

8q24.13	8:126500031:C:G	8	126435663	126533955	rs28601761	1.83E–18	RP11–136O12.2	0.983

19p13.11	19:19379549:C:T	19	19260760	19865077	rs58542926	2.87E–13	TM6SF2	0.766

19q13.32	19:45415713:A:G	19	45332635	45428234	rs10414043	3.61E–13	APOE, APOC1	0.854

22q13.31	22:44341986:C:T	22	44323370	44411044	rs2294917	2.90E–08	PNPLA3	0.090


The pleiotropic results were analyzed by MAGMA gene set enrichment analysis, and the results showed that the top 10 significant gene sets were enriched (Table 2). This includes gene sets such as GOBP _ POSITIVE _ REGULATION _ OF _ EPITHELIAL _ CELL _ MIGRATION, PAL _ PRMT5 _ TARGETS _ UP and GOBP _ POSITIVE _ REGULATION _ OF _ ENDOTHELIAL _ CELL _ MIGRATION. Tissue-specific MAGMA analysis of 30 common tissues showed that the two diseases were significantly enriched in liver tissues (Figure S10). Further performed enrichment analysis on 54 tissues (Figure S11). Among these 54 tissues, the liver is the most specific, followed by the esophagus gastroesophageal junction, lung and artery coronary. It is worth noting that this part of MAGMA gene set and tissue specificity analysis were analyzed using the complete distribution of SNP *P* values.

**Table 2:** Gene set analysis results (top 10).


GENE SET	N GENES	BETA	SE	P	PADJ

GOBP_POSITIVE_REGULATION_OF_EPITHELIAL_CELL_MIGRATION	144	0.320	0.075	1.02E–05	0.101

PAL_PRMT5_TARGETS_UP	186	0.248	0.061	2.35E–05	0.101

GOBP_POSITIVE_REGULATION_OF_ENDOTHELIAL_CELL_MIGRATION	101	0.350	0.087	2.95E–05	0.101

GOBP_AMEBOIDAL_TYPE_CELL_MIGRATION	407	0.174	0.044	3.56E–05	0.101

GOBP_NOREPINEPHRINE_UPTAKE	8	1.448	0.365	3.74E–05	0.101

GOBP_REGULATION_OF_EPITHELIAL_CELL_MIGRATION	216	0.236	0.061	5.05E–05	0.101

PID_ANGIOPOIETIN_RECEPTOR_PATHWAY	46	0.479	0.123	5.19E–05	0.101

GOBP_NEGATIVE_REGULATION_OF_MUSCLE_ADAPTATION	9	1.290	0.334	5.68E–05	0.101

ZHAN_MULTIPLE_MYELOMA_CD1_DN	42	0.518	0.138	8.82E–05	0.121

GOBP_POSITIVE_REGULATION_OF_MONOCYTE_DIFFERENTIATION	9	1.109	0.297	9.63E–05	0.125


**Figure 2:**
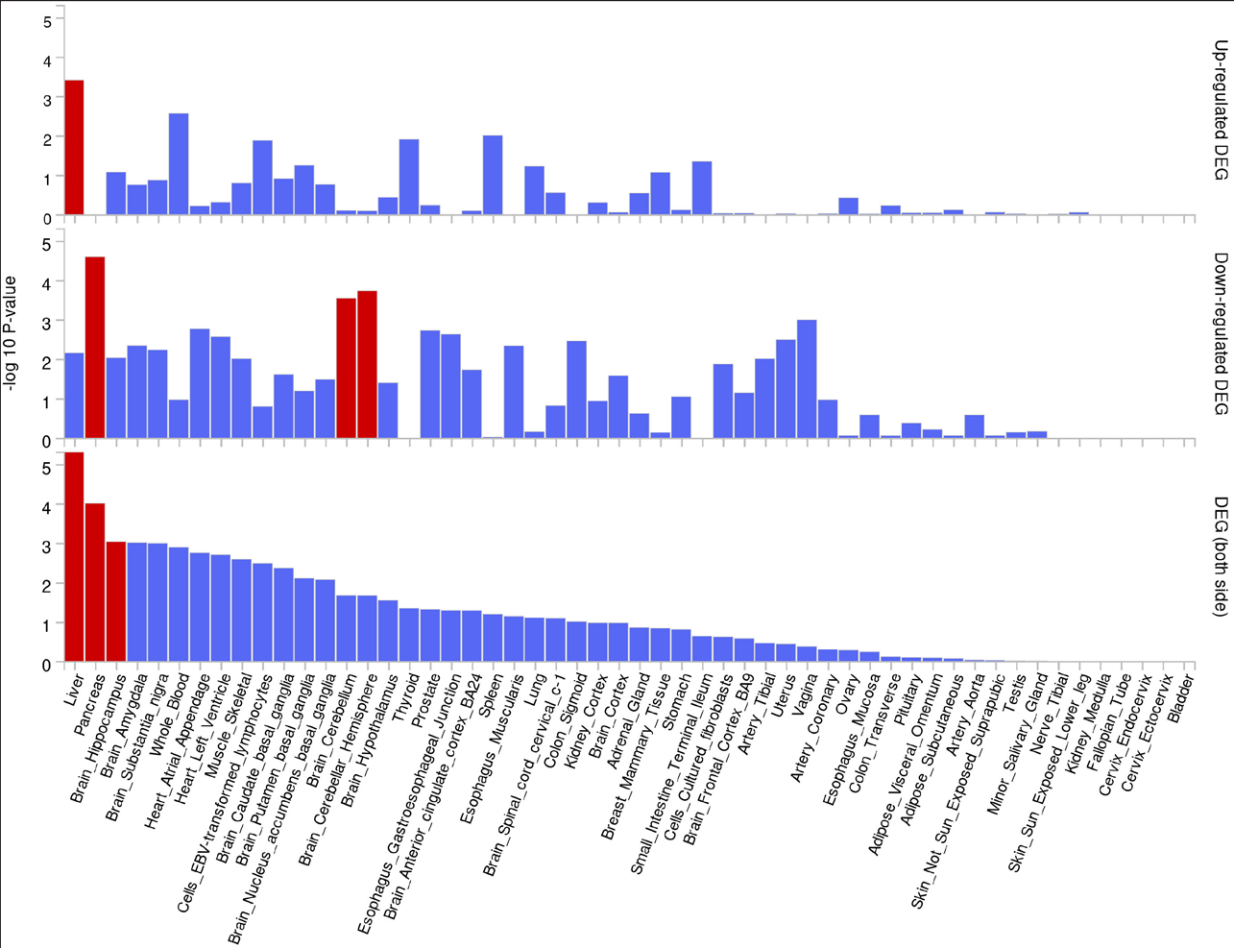
Enrichment of pleiotropic genes in different tissues.

**Figure 3:**
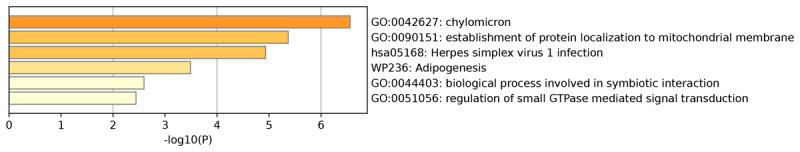
Pathway enrichment of pleiotropic eQTL genes (KEGG, wiki, GO).

Through the location information of the lead SNP, we matched 7 nearby genes (IGF2R, AC100802.3, RP11-136O12.2, TM6SF2, APOE, PNPLA3 and APOC1) associated with these pleiotropic risk loci (Table 1). The mAGMA gene analysis identified three pleiotropic genes (APOC1, TOMM40 and PBX4) (Figure S12, Table S4); for the QQ diagram, see Figure S13. Further using eQTL information (data of whole blood, liver, heart and vascular tissue), we matched eQTL genes associated with these pleiotropic risk loci (Table S5). All pleiotropic genes (nearby gene + MAGMG gene + eQTL gene, Table S6) showed significant differential expression in the liver, EBV-transformed lymphocytes, whole blood and other tissues (Figure S14 and Table S8). The enrichment in different tissues is shown in Figure 2 and Table S7. The main enriched tissues were the liver, pancreas and brain hippocampus. Pathway enrichment analysis is shown in Figure 3. Pathway enrichment includes chylomicron, establishment of protein localization to the mitochondrial membrane and herpes simplex virus 1 infection. And cell-specific enrichment analysis is shown in Figure S15, for example, DURANTE ADULT OLFACTORY NEUROEPITHELIUM OLFACTORY ENSHEATHING GLIA, FAN OVARY CL5 HEALTHY SELECTABLE FOLLICLE THECAL CELL and TRAVAGLINI LUNG MACROPHAGE CELL.

### No causal relationship between NAFLD and CAD

**Figure 4:**
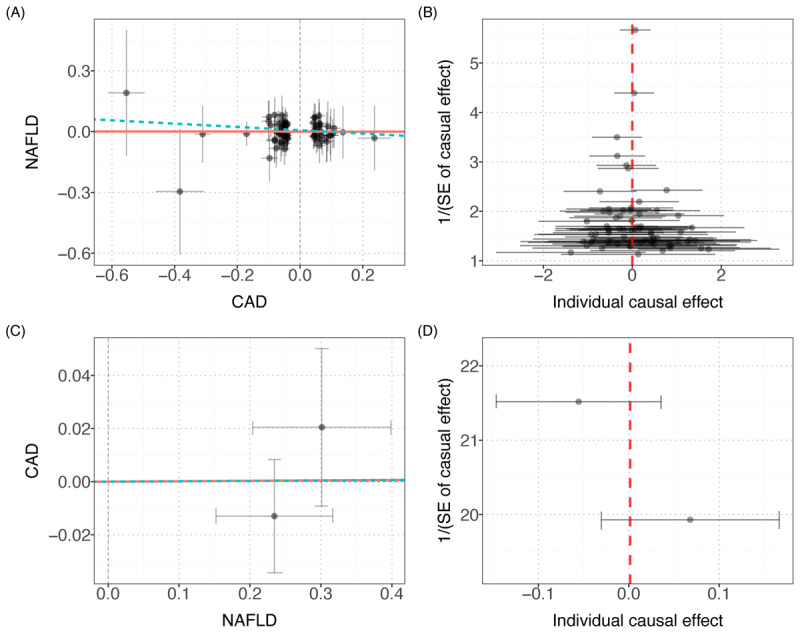
Scatter plot and funnel plot of MR analysis. A: the scatter plot of the causal effect of CAD on NAFLD. B: the funnel plot of the causal effect of CAD on NAFLD. C: the scatter plot of the causal effect of NAFLD on CAD. D: the funnel plot of the causal effect of NAFLD on CAD.

Finally, the two-sample MR method was used to infer the causal relationship between the two diseases, and the results did not support the previous significant correlation between the two. The sensitivity analysis is shown in Table 3, and the scatter plot and funnel plot are shown in Figure 4.

**Table 3:** The results of MR analysis.


EXPOSURE	OUTCOME	METHODS	ESTIMATE	*P*	HETEROGENEITY TEST	

					ESTIMATE	*P*

NAFLD	CAD	IVW (fixed)	1.002 (0.937,1.071)	0.964	3.254	0.071

		IVW (random)	1.002 (0.888,1.13)	0.980		

		DIVW	1.002 (0.917,1.094)	0.972		

		MR–RAPS	1.002 (0.936,1.072)	0.963		

CAD	NAFLD	MR–RAPS	0.999 (0.884,1.129)	0.990	79.260	0.187

		IVW (fixed)	0.999 (0.877,1.139)	0.991		

		IVW (random)	0.922 (0.697,1.218)	0.562		

		MR–Egger (slope)	0.007 (–0.013,0.027)	0.512		

		MR–Egger (intercept)	0.961 (0.741,1.245)	0.761		

		Weighted mode	1.001 (0.821,1.22)	0.994		

		Weighted median	0.999 (0.876,1.14)	0.991		

		DIVW	0.999 (0.883,1.131)	0.990		

		MR–RAPS	1.002 (0.937,1.071)	0.964		


## Discussion

NAFLD and CAD have been major public health problems affecting millions of people around the world. Current studies suggest that these two diseases are the result of complex interactions between genetics, diet and environment, but their pathogenesis has not been fully elucidated. In recent years, with more and more studies on the correlation between NAFLD and coronary heart disease gene polymorphisms, it may provide new methods for the diagnosis, prevention and treatment of these two diseases (6).

In the current study, we found a significant genetic overlap between NAFLD and CAD. In addition, we identified six pleiotropic loci between NAFLD and CAD and identified apolipoprotein C1 (APOC1), translocase of outer mitochondrial membrane 40 (TOMM40) and PBX Homeobox 4 (PBX4) as shared risk genes by MAGMA gene analysis. At the same time, MR analysis showed that there was no causal relationship between NAFLD and CAD. These results suggest that there is a common genetic structure and potential common pathogenesis between NAFLD and CAD, which provides a better understanding of the pleiotropic effects of NAFLD and has guiding significance for the clinical treatment of related complex phenotypes.

We first analyzed the genetic correlation between NAFLD and CAD and found a significant positive genetic correlation between NAFLD and CAD. This is consistent with previous observational studies finding comorbidities between NAFLD and CAD (31). However, the results should be interpreted with caution due to the differences in the database selection. At the same time, the ethnic differences in the database will reduce the accuracy of analyzing the genetic correlation. Further analysis of GWAS based on larger sample sizes and multiple populations is still necessary.

We observed substantial genetic overlap between NAFLD and CAD. Previous studies have identified shared genetic architecture between NAFLD and CAD and used similar approaches to identify genetic enrichment and shared risk loci (17,32). Our study identified a significant correlation between NAFLD and CAD from both genetic and transcriptional perspectives. Furthermore, with the MR approach, we identified a common etiology between NAFLD and CAD, which further suggests a potential common etiology between these two diseases. These multiple associations suggest that there may be a common pathogenesis or genetic background between NAFLD and CAD, which warrants further exploration. However, since this association is nominally significant, the causal relationship should be interpreted with caution.

Using the MAGMA gene analysis, we identified three common risk genes, namely, APOC 1, TOMM40, and PBX 4. Selvarajan et al. (33) used liver cis-eQTL analysis and promoter capture in HepG 2 cells to identify Hi-C and identified susceptibility enhancers near important cholesterol homeostasis genes (APOC 1), suggesting that altered gene regulatory activity may represent another way of genetic variation to regulate serum lipoprotein levels and to identify putative causal regulatory regions and target genes that may affect liver function and susceptibility to clinical manifestations of coronary disease. TOMM40 Sigis attributed to APOE RS 429358-T, while APOE allele-specific variants increase nonalcoholic fatty liver disease and obesity but decrease the risk of Alzheimer’s disease and myocardial infarction (34). For CAD, some studies have found that TOMM40 is a locus significantly associated with non-HDL-C (35), and some studies have incorporated TOMM40 into the composition of the longevity model (36). For PBX 4, our newly identified risk gene, no studies have analyzed its role between NAFLD and CAD. It should be noted that other tissues may have been missed because we focused on the enrichment analysis of liver tissue. With further understanding of the relevant tissues of NAFLD and CAD, further exploration of these tissues is still necessary.

We also performed functional enrichment analysis using pleiotropic genes and identified several enriched pathways that somehow participate in the pathogenesis of NAFLD and CAD, which deserve attention in future studies. Finally, we found that there is no positive or negative causal relationship between NAFLD and CAD. Surprisingly, this result is contrary to several studies (17,37) and the same as some studies (38). We consider that the reason for the inconsistent results may be the difference in the selected databases and the difference in the genetic instrumental variables of MR used.

This study has limitations. First, we found that there are multiple pleiotropic genes and loci between NAFLD and CAD, which requires further in-depth experimental studies to determine whether they directly affect the phenotypes studied and the causal mechanisms involved. In addition, the populations studied in this study are all of European origin and need to be further expanded to other populations for verification. Finally, because we are based on a pooled population-level study, there is no individual-level data, so there is no way to conduct age, gender and other stratified studies, which may affect our results.

For the recommendations of future studies, first, further understanding of the genetic structure and pleiotropic gene loci that regulate the overlap between NAFLD and CAD may involve the pathophysiological processes of NAFLD and CAD and may form the basis for preventive measures and identification of new targets for drug development. In addition, we identified three pleiotropic genes. For example, with APOC 1, previous studies have been associated with NAFLD and not with CAD, but we found that it is also related to them. At this time, APOC 1 is a new target. Secondly, this is the potential direction of future animal research and large-scale comorbidity or specific disease cohort studies.

## Conclusions

Through the LDSC and SNP-Level PLACO methods, we evaluated the significant genetic correlation between NAFLD and CAD and identified selective pleiotropy and new shared loci between them. Combined with magmatic gene analysis, we further identified the common pleiotropic genes APOC1, TOMM40 and PBX4. MR analysis showed that there was no nominal causal relationship between NAFLD and CAD. These findings may provide new insights into the genetic overlap between NAFLD and CAD and help to better understand their etiology.

## Data Accessibility Statement

The authors confirm that the data supporting the findings of this study are available within the article and its supplementary materials.

## Additional File

The additional file for this article can be found as follows:

10.5334/gh.1374.s1 Supplementary Material. Figure S1 to S15.

## Abbreviations

NAFLD, nonalcoholic fatty liver disease; CAD, coronary artery disease; LDSC, linkage disequilibrium score regression; MR, mendelian randomization; MS, metabolic syndrome; HCC, hepatocellular carcinoma; CVD, cardiovascular disease; PPAR, peroxisome proliferator-activated receptors gene; LEPR, leptin receptor; APOC3, apolipoprotein C3; IUT, implementation under test; MAGMA, generalized Gene-Set Analysis of GWAS Data; FUMA, functional maps and annotations; MSigDB, molecular signatures database; IVW, inverse variance weighting; APOC1, apolipoprotein C1; TOMM40, translocase of outer mitochondrial membrane 40; PBX 4, PBX Homeobox 4.
