# Supplementary material for: Shared Genetic Links Between Nonalcoholic Fatty Liver Disease and Coronary Artery Disease
Source: Glob Heart. 2024 Nov 26;19(1):88. doi: 10.5334/gh.1374 (PMC11606392; doi:10.5334/gh.1374)
Supplement: Supplementary Material. — Figure S1 to S15. [file gh-19-1-1374-s1.pdf]

Supplementary Material

Figure S1. QQ plot for pleiotropic analysis.

Figure S2. Summary of basic information for each genomic risk locus.

Figure S3. Effects of pleiotropic SNP on gene function.

Figure S4. Regional plot of the locus (6q25.3).

Figure S5. Regional plot of the locus (8p21.3).

Figure S6. Regional plot of the locus (8q24.13).

Figure S7. Regional plot of the locus (19p13.11).

Figure S8. Regional plot of the locus (19q13.32).

Figure S9. Regional plot of the locus (22q13.31).

Figure S10. MAGMA tissue-specific analysis based on genome-wide pleiotropic (30 common GTEx tissues).

Figure S11. MAGMA tissue specificity analysis based on genome-wide pleiotropic (54 GTEx tissues).

Figure S12. MAGMA Gene Analysis Manhattan Chart.

Figure S13. The QQ plot of MAGMA gene analysis.

Figure S14. Expression of pleiotropic genes in different tissues.

Figure S15. Cell-type enrichment of pleiotropic eQTL genes.

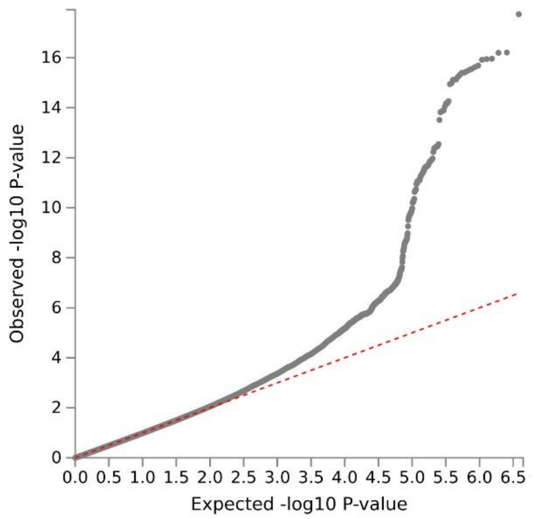

Figure S1. QQ plot for pleiotropic analysis.

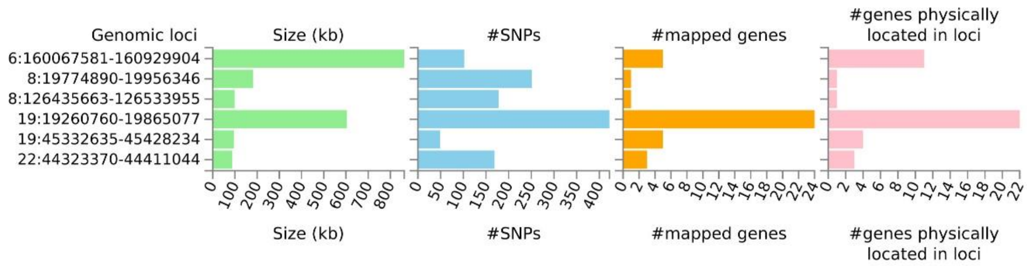

Figure S2. Summary of basic information for each genomic risk locus.

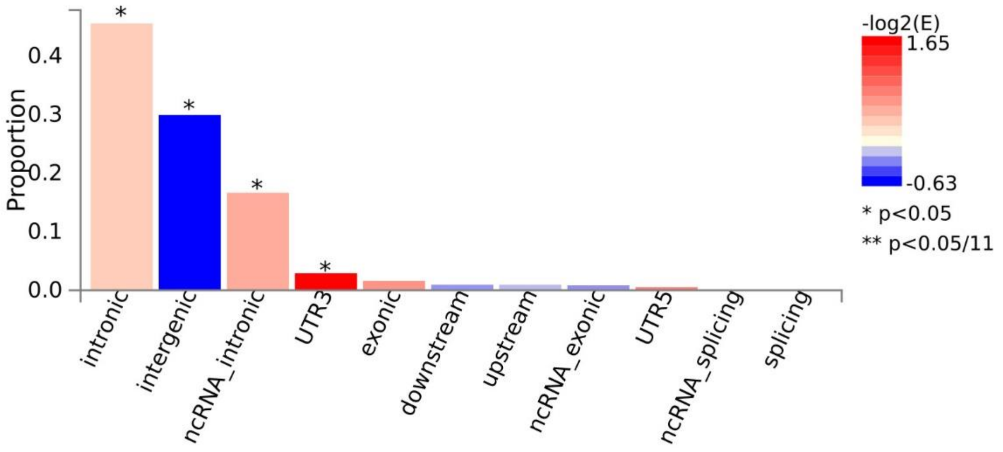

Figure S3. Effects of pleiotropic SNP on gene function.

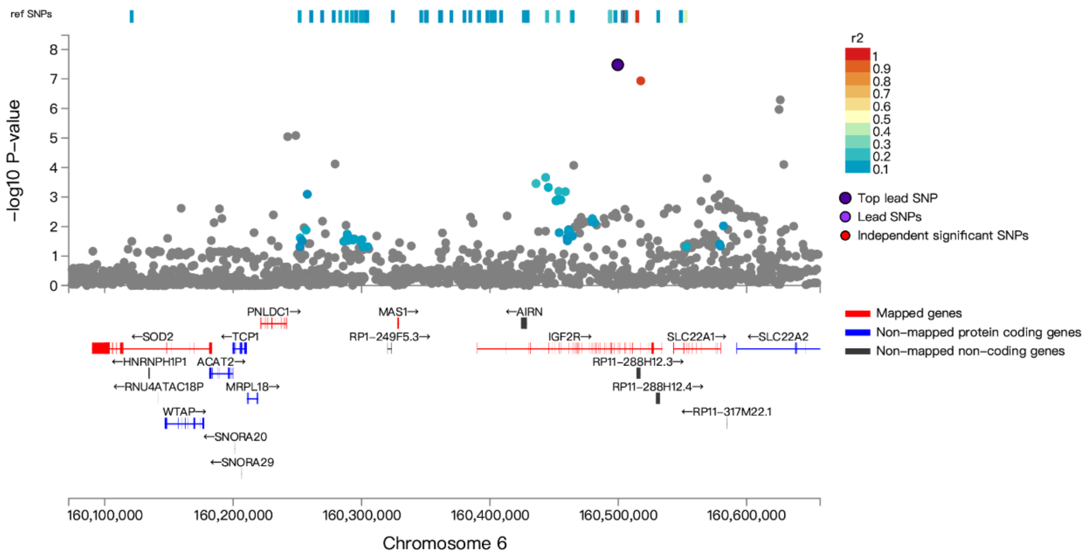

Figure S4. Regional plot of the locus (6q25.3).

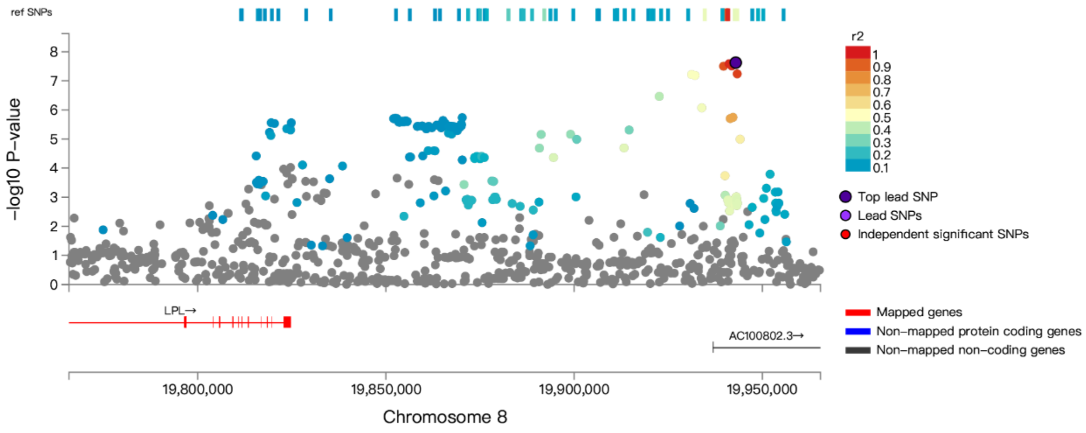

Figure S5. Regional plot of the locus (8p21.3).

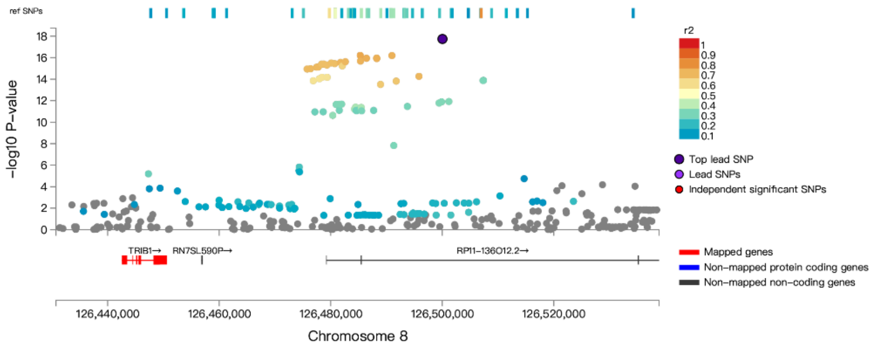

Figure S6. Regional plot of the locus (8q24.13).

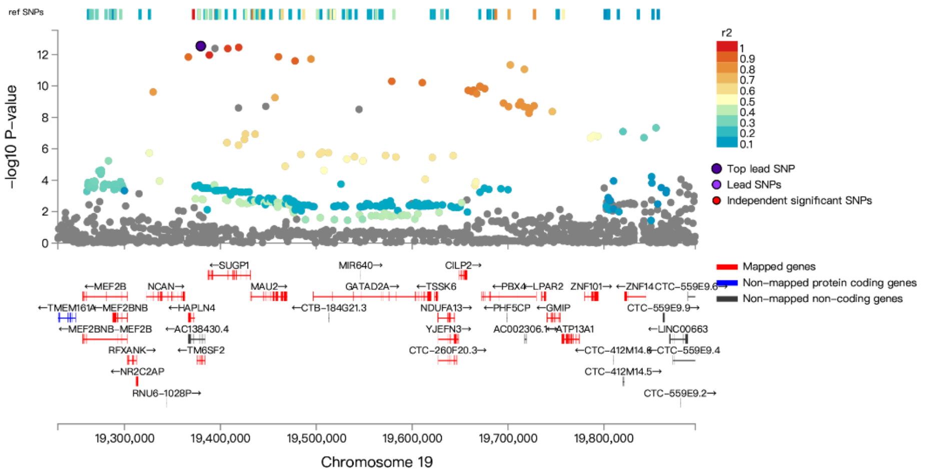

Figure S7. Regional plot of the locus (19p13.11).

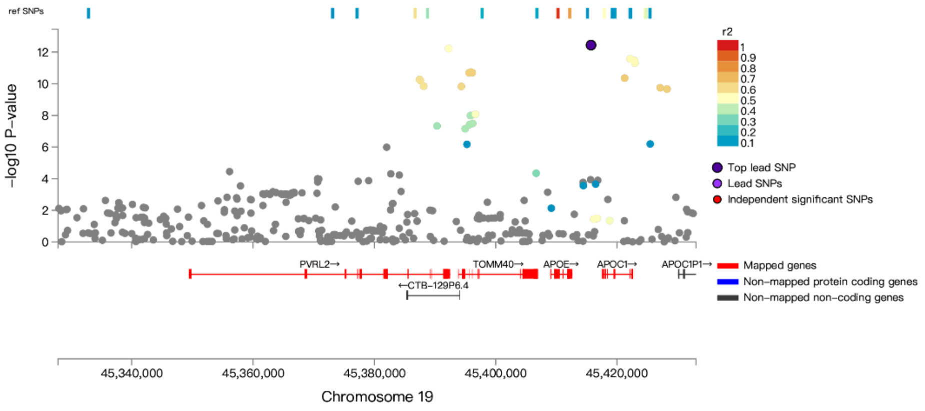

Figure S8. Regional plot of the locus (19q13.32).

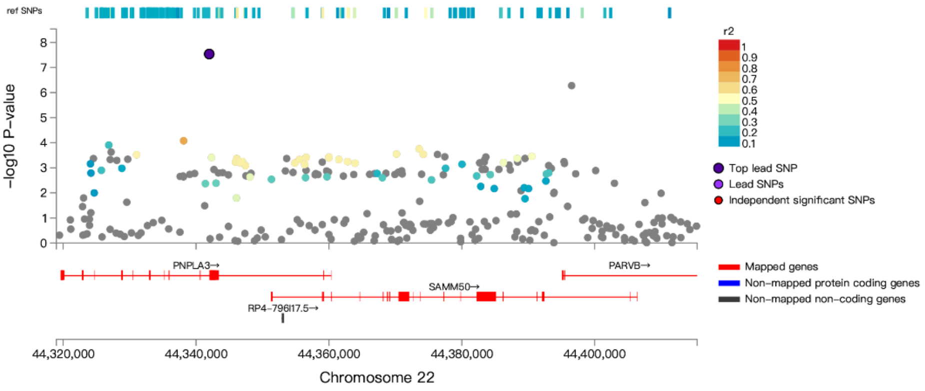

Figure S9. Regional plot of the locus (22q13.31).

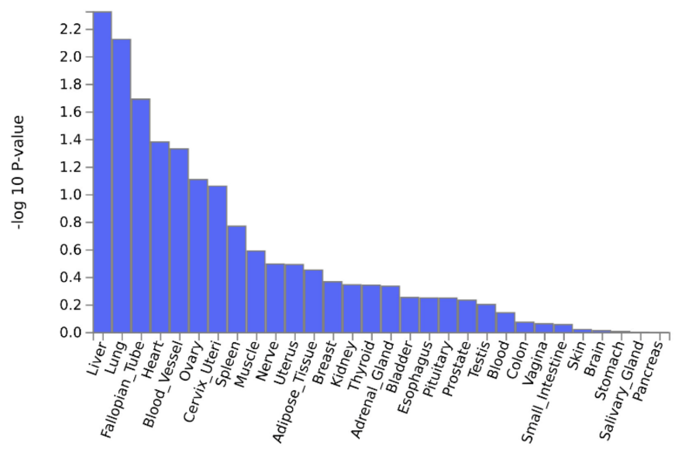

Figure S10. MAGMA tissue-specific analysis based on genome-wide pleiotropic (30 common GTEx tissues).

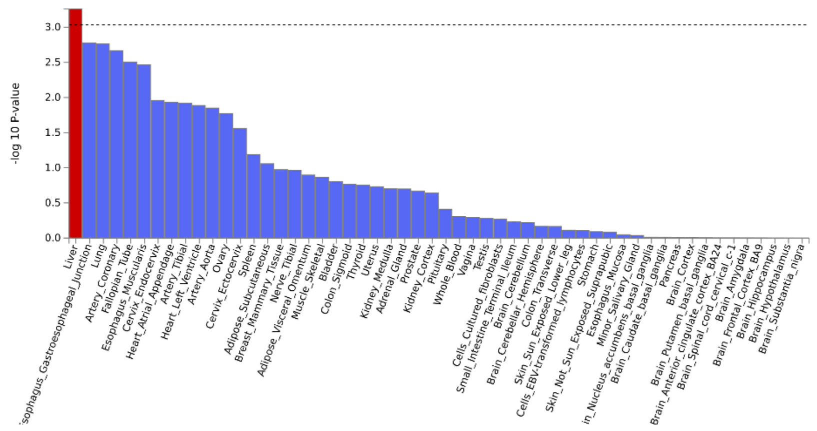

Figure S11. MAGMA tissue specificity analysis based on genome-wide pleiotropic (54 GTEx tissues).

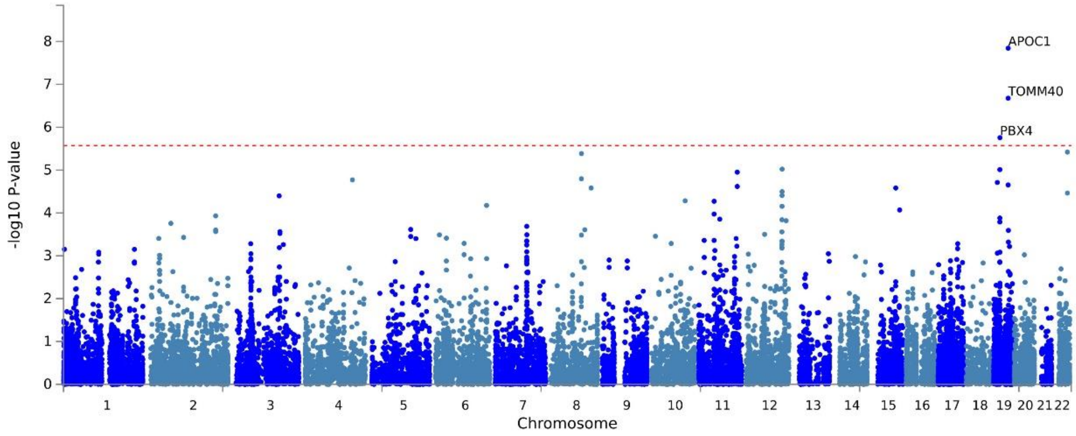

Figure S12. MAGMA Gene Analysis Manhattan Chart.

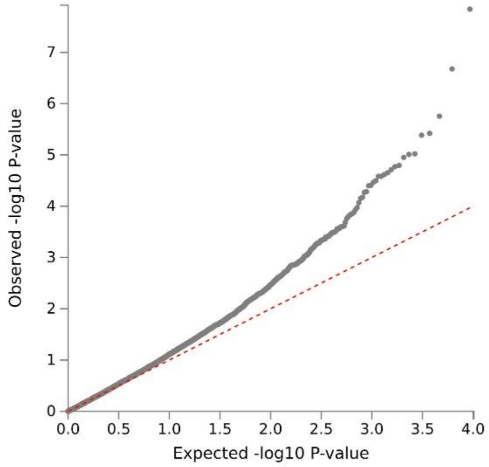

Figure S13. The QQ plot of MAGMA gene analysis.

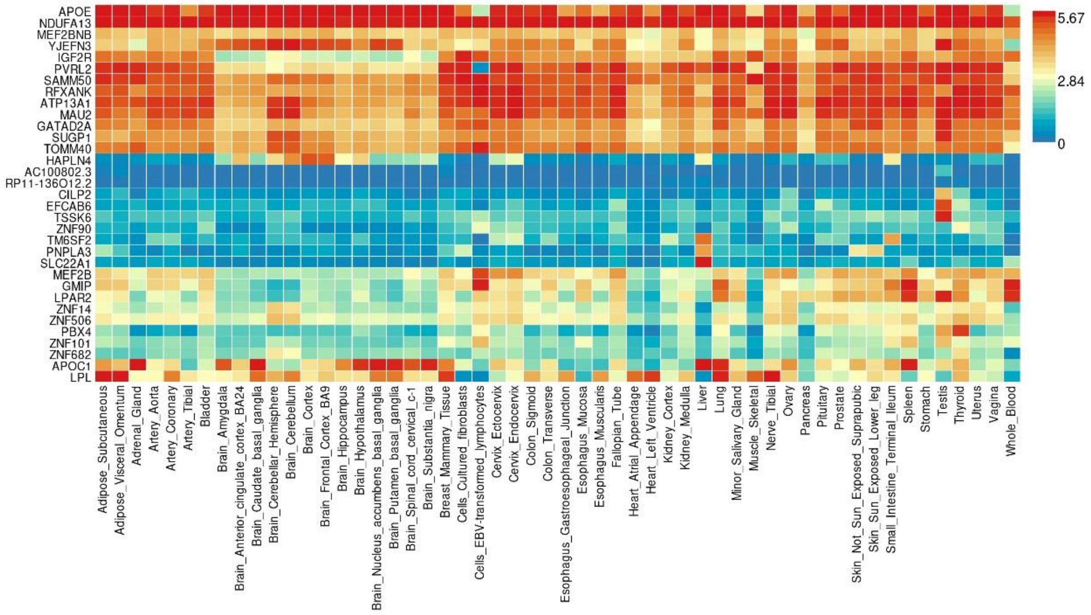

Figure S14. Expression of pleiotropic genes in different tissues.

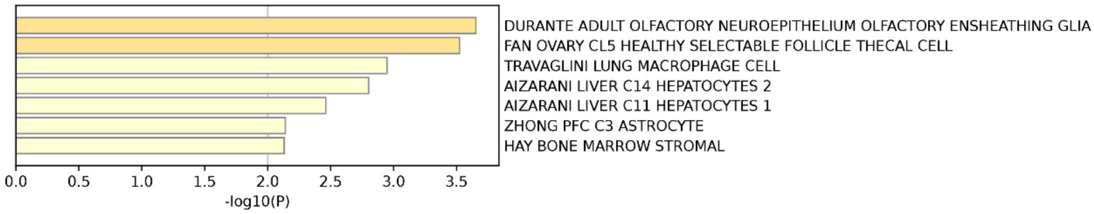

Figure S15. Cell-type enrichment of pleiotropic eQTL genes.
